# Annals of Gynecology

**Published:** 1887-10

**Authors:** 


ANNALS OF GYMNÆCOLOGY, VOL. 1, No.1, OCTOBER '87, a monthly review of Gymnæcology, Obstetrics and Abdominal Surgery, edited by E. W. Cushing, M. D., Boston, with the collaboration of Prof. T. G. Thomas, M. D., Prof. W. G. Wylie, M. D., Prof. W. K. Polk, M. D., Prof. DeLaskie Miller, M. D., Chicago, Dr. Andrew F. Currier, New York. Published by Rockwell & Churchill, Boston, price first year, $1; after that, $3.

We acknowledge receipt of Vol. 1, No. 1, of the above new journal, and place it on our exchange list wlth pleasure.

